# Spatiotemporal epidemic characteristics and risk factor analysis of malaria in Yunnan Province, China

**DOI:** 10.1186/s12889-016-3994-9

**Published:** 2017-01-11

**Authors:** Dongyang Yang, Chengdong Xu, Jinfeng Wang, Yong Zhao

**Affiliations:** 1 School of Geographic Sciences, East China Normal University, Shanghai, 200241 China; 2 State Key Laboratory of Resources and Environmental Information System, Institute of Geographic Science and Natural Resource Research, Chinese Academy of Sciences, Beijing, 100101 China; 3 Key Laboratory of Surveillance and Early Warning on Infectious Disease, Chinese Center for Disease Control and Prevention, Beijing, 102206 China; 4 College of Environment and Planning, Henan University, Kaifeng, 475004 Henan China

**Keywords:** Malaria, Epidemiologic characteristic, Space-time clusters, Risk factors

## Abstract

**Background:**

Malaria remains an important public health concern in China and is particularly serious in Yunnan, a China’s provincial region of high malaria burden with an incidence of 1.79/10^5^ in 2012. This study aims to examine the epidemiologic profile and spatiotemporal aspects of epidemics of malaria, and to examine risk factors which may influence malaria epidemics in Yunnan Province.

**Methods:**

The data of malaria cases in 2012 in 125 counties of Yunnan Province was used in this research. The epidemical characteristics of cases were revealed, and time and space clusters of malaria were detected by applying scan statistics method. In addition, we applied the geographically weighted regression (GWR) model in identifying underlying risk factors.

**Results:**

There was a total of 821 cases of malaria, and male patients accounted for 83.9% (689) of the total cases. The incidence in the group aged 20–30 years was the highest, at 3.00/10^5^. The majority (84.1%) of malaria cases occurred in farmers and migrant workers, according to occupation statistics. On a space-time basis, epidemics of malaria of varying severity occurred in the summer and autumn months, and the high risk regions were mainly distributed in the southwest counties. Annual average temperature, annual cumulative rainfall, rice yield per square kilometer and proportion of rural employees mainly showed a positive association with the malaria incidence rate, according to the GWR model.

**Conclusions:**

Malaria continues to be one of serious public health issues in Yunnan Province, especially in border counties in southwestern Yunnan. Temperature, precipitation, rice cultivation and proportion of rural employees were positively associated with malaria incidence. Individuals, and disease prevention and control departments, should implement more stringent preventative strategies in locations with hot and humid environmental conditions to control malaria.

## Background

Malaria is one kind of mosquito-borne communicable diseases caused by the genus *Plasmodium*, and transmitted in both humans and other animals. It is the world’s most serious insect-borne disease with the highest rate in morbidity and mortality among all vector-borne infectious diseases. In 2015, there was a total of 95 countries and territories in which malaria was considered endemic. The estimated number of population who live in risk areas of malaria is 3.2 billion, and the World malaria report 2015 estimates that 214 million malaria cases occurred globally in 2015, resulting in 438,000 deaths [1]. In tropical and subtropical regions, such as Southeast Asia, Sub Saharan Africa, and Eastern Mediterranean, the disease is widespread. In China, malaria is on the list of category B notifiable diseases to be reported according to the law on prevention and treatment of infection diseases in China. Although the incidence of malaria has decreased recently, it remains an important public health problem in China [2].

Yunnan Province, located in southwest China, is a province that records serious malaria outbreaks and epidemics in China [3–7]. According to national network surveillance data, the cases of malaria in Yunnan Province respectively accounted for 31.4, 14.0 and 17.3% of the overall cases in 2012, 2013 and 2014 in this country. The reasons for the serious malaria epidemics in Yunnan Province may include its muggy tropical and subtropical climate, especially in tropical valleys [8, 9], and social factors such as the international movement of individuals to and from Laos, Myanmar and Vietnam. The trans-national population movement has been a major obstacle of effective malaria prevention and repetitive epidemics [10, 11].

A specific understanding on the spatial and temporal characteristics of malaria epidemics may lead to effective resource allocation and intervention activities. Regional differences in malaria epidemics are related to environmental conditions, which play a vital part in the breeding and population size of *Anopheles* mosquitoes, and on malaria transmission [12]. With complex environmental conditions, this region was seen various common *Anopheles* species, such as *Anopheles minimus*, *Anopheles kunmingensis*, *Anopheles lesteri anthropophagus*, *Anopheles sinensis* and *Anopheles dirus* [13, 14]. Rice fields and their surrounding area, such as irrigation canals, were the common breeding grounds except for *Anopheles dirus*, and all of these *Anopheles* species have a strong human-biting habit. Climate warming has caused an extension of the malaria transmission season, and has triggered research into the relationship between malaria and meteorological factors [15–17].

In this study, we analyzed the epidemic characteristics of malaria in Yunnan Province based on the data from cases that occurred during 2012, conducted space-time scan analysis to detect time and space clusters, and examined the risk factors for malaria by applying a geographically weighted regression (GWR) model. In 2010, Chinese government launched a national campaign on malaria elimination, removing the disease throughout China by 2020 was targeted [18]. This study may provide information for the development of new policy and measures in the implementation of actions to eliminate malaria.

## Methods

### Study area

**Fig. 1:**
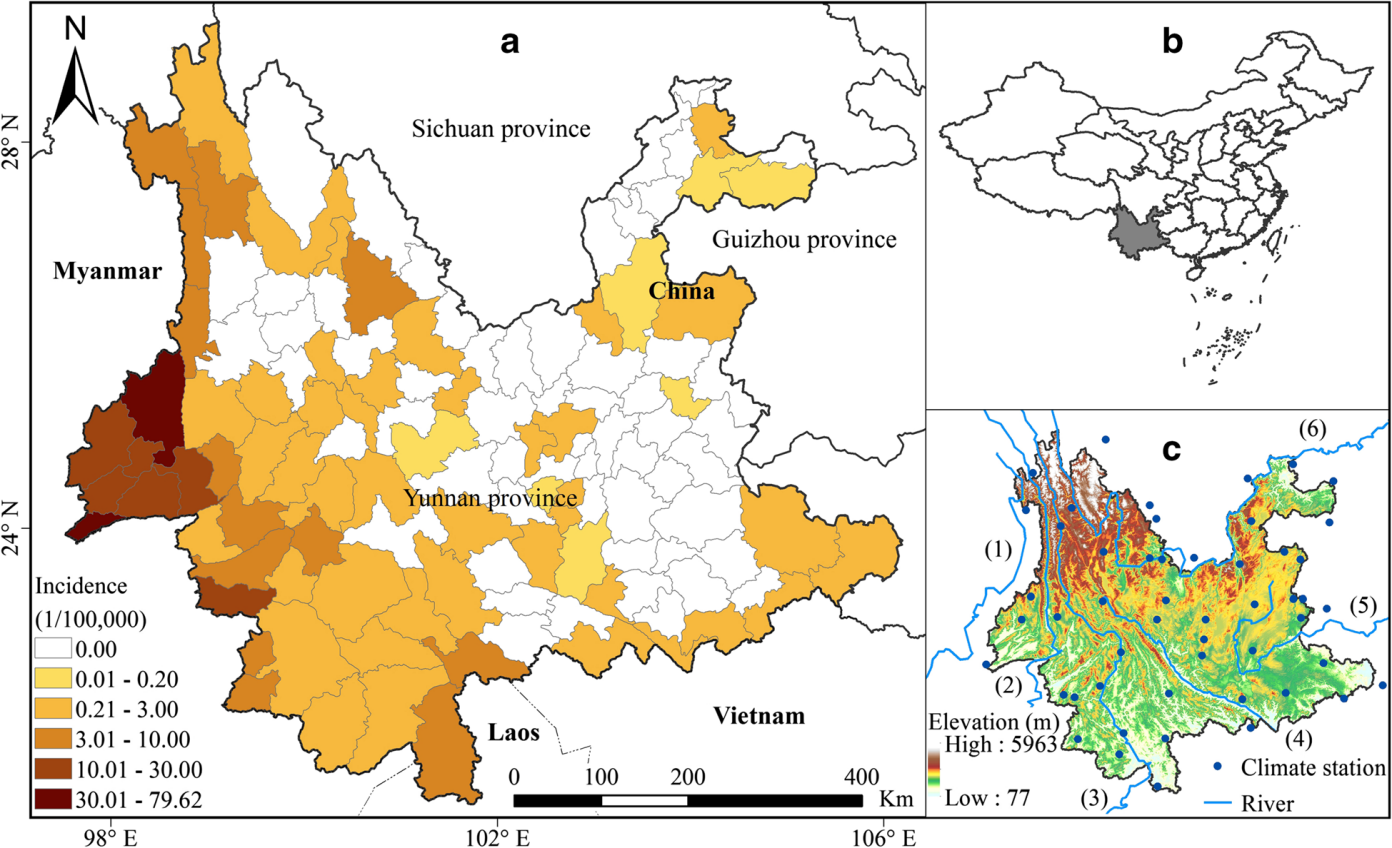
Yunnan province and its malaria incidence (**a**), Geographic location in China (**b**), topography and distribution of vicinal meteorological stations (**c**)

Yunnan Province is situated on the Yunnan-Guizhou Plateau in China’s south-western frontier region, and has a complex geography. The province covers an area of over 390,000 km^2^, with a population of 45,966,239 (China population census 2010), and is adjacent to Myanmar, Laos, and Vietnam with a 4,060 km border (Fig. 1). Located in the middle and low latitudes, Yunnan has a humid monsoon climate typical of the tropical and subtropical plateau zone. The annual precipitation is about 1100 mm in most parts of Yunnan, but more than 1600 mm in the southern regions. The topography is lower in the south and higher in the north with great terrain height difference, which exacerbates the temperature difference from south to north. The annual temperature is about 5 °C–24 °C with a south-north temperature difference reaching about 19 °C. Rice is one of the main food crops; rice cultivation occurs in all counties of Yunnan. Six major rivers flow through this area, namely: Irrawaddy, Salween, Mekong, Red River, Pearl River and Jinsha River.

### Malaria surveillance data

Malaria is a notifiable disease in China, and the records of malaria cases in the region from January to December 2012 were obtained from hospital reports. Data on case demographic informations, location, onset date, etc. were aggregated for each county. The research data involved a total of 125 counties (cities or states) of Yunnan Province.

### Socioeconomic and meteorological data

Data on the total population, employed population and rice production in 2012 for each county were obtained from the Yunnan Statistical Yearbook. The proportion of rural employees is the percentage of the number of rural employees in total population, the number of rural employees for each county were obtained from China County Statistical Yearbook (the number of rural employees for five municipal districts, including Kunming, Qujing, Yuxi, Baoshan and Lijiang were the estimations of the agricultural population). The incidence was based on cases of malaria and the total population. We estimated the annual incidence in each county in order to realize the spatial profile of malaria in Yunnan (Fig. 1). Meteorological data, including the annual average temperature and annual cumulative rainfall data for each county, were obtained by interpolating annual meteorological station values, which were available online in the China Meteorological Data Sharing Service System (http://data.cma.cn/). The climatological stations in Yunnan province and nearby this region were mapped in Fig. 1 to show their spatial distribution.

### Spatial-temporal cluster analysis

Spatial-temporal clusters of malaria cases were identified using the space-time scan statistic incorporated in SaTScan version 9.4 (www.satscan.org). A columniform window of variable size was used to define the space-time scan statistic. The bottom and the height of the window corresponds to geographic extent and time respectively. The potential spatial-temporal clusters, which are the periods and geographical areas with the highest risk, would be detected along with the dynamic changes of the bottom and the height of the window [19]. The center of bottom is the centroid of one or multiple spatial units in the research area. The population size who may be at risk within a variable number of spatial units was used to define the radius of the window.

The center and radius of the bottom and the height are constantly changing; the default maximum radius is 50% of the population within the study region. Each time they change, the software calculates the log likelihood ratio (*LLR*) to compare the internal and the external incidence of the window. The formula is:

$$ LLR= \log \left\{{\left(\frac{n_c}{n_e}\right)}^{n_c}{\left(\frac{N_c-{n}_c}{N_c-{n}_e}\right)}^{\left({N}_c-{n}_c\right)}\right\} $$

where *n*_*c*_ denotes the number of cases in a window. The expected number of cases in the window and the total cases number are denoted by *n*_*e*_ and *N*_*c*_ respectively. Each of the windows has a *LLR* value, the candidate clusters are defined by the windows with statistically significant *LLR*. The most likely cluster is the window which has the maximum *LLR*, and the secondary clusters are defined by other scan windows. Monte Carlo simulation is used to evaluate the *p*-value in conducting the scan statistic [20].

The 125 counties in Yunnan were the spatial units, month was the scan time scale in conducting the space-time scan statistic in this study.

### Geographically weighted regression

The GWR model is an extension of the traditional regression model used to detect spatial non-stationarity of parameters. A spatial weights matrix is added to estimate local parameters. The formula of GWR is:

$$ {Y}_i={\beta}_0\left({u}_i,{v}_i\right)+{\displaystyle \sum_m{\beta}_m\left({u}_i,{v}_i\right){X}_{im}+{\varepsilon}_i\;i=1,\cdots, n} $$

where (*u*_*i*_, *v*_*i*_) is the longitude and latitude of observation position *i*, *β*_0_(*u*_*i*_, *v*_*i*_) is the local constant. *β*_*m*_(*u*_*i*_, *v*_*i*_) are the parameters which change with geographical location. Thus, in this model, the local effects could be captured as the parameters are estimated across space [21].

Locally weighted least squares analysis is used to calibrate the model. The regression parameters of point *i* are obtained by local regression of observations of immediate neighbors, as the spatial correlation hypothesis indicates that geographic elements at close range have a greater correlation than the elements at a greater distance. The local parameters *β*_*m*_(*u*_*i*_, *v*_*i*_) is calculated by the following equation,

$$ \widehat{\beta}\left({u}_i,{v}_i\right)={\left[{X}^TW\left({u}_i,{v}_i\right)X\right]}^{-1}{X}^TW\left({u}_i,{v}_i\right)Y $$

where *W*(*u*_*i*_ *,v*_*i*_) is the spatial weights matrix which is used to present the spatial neighboring relationships between observation *i* and its surrounding points. The weight matrix is computed for each point *i* at which parameters are estimated. By applying the GWR model, the local effects of factors may be assessed, which is not possible using traditional ordinary least squares (OLS) methods [22].

## Results

### Epidemiologic characteristics

**Fig. 2:**
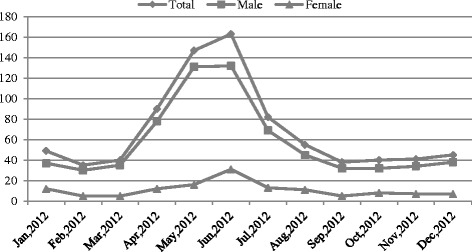
Changes of occurrence of malaria cases by month in Yunnan province during year 2012

A total of 821 malaria cases occurred in Yunnan Province during the whole of 2012. Male patients accounted for 83.9% (689) of all malaria cases, and the incidence in males was 2.89/10^5^, significantly higher than that in females (0.60/10^5^). The group aged 30–40 years accounted for the largest proportion of all reported malaria cases: 27.4% (225). The incidence in group 20–30 years was the highest (3.00/10^5^), followed by the incidence in those aged 30–40 years (2.72/10^5^). The majority (approximately 67.4%) of malaria cases occurred in farmers, according to occupational statistics. The distribution of the submitted malaria cases is presented in Table 1. The malaria cases showed obvious seasonal variation over time. Most of these cases were concentrated in the summer, when the weather was hot and humid, especially in places with lush vegetation, high mountains and deep valleys. Males accounted for most of the total (83.9%), while cases in females were rare. Therefore, the cases in male patients mirrored the month-by-month changes of the total cases, with a slight decrease from January to February, a rapid rise from March, a peak of 132 cases in June, and a rapid decrease from June to September. The lowest number of cases in males occurred in February (30 cases). The number of female cases changed little throughout the year, with a peak of 31 cases in June and fewer than 20 cases in other months. The lowest number of total cases occurred in February (35 cases), and the highest number occurred in June, when the total number of cases was 163 (Fig. 2).

**Table 1:** Descriptive statistics for the malaria cases of Yunnan Province during the year 2012

Category		Percentage (%)	Incidence (per 10^5^)	No of malaria infections
Sex	Male	83.9	2.89	689
	Female	16.1	0.60	132
Age	<5	1.1	0.30	9
	5–10	2.6	0.67	21
	10–20	13.4	1.52	110
	20–30	26.8	3.00	220
	30–40	27.4	2.72	225
	40–50	20.8	2.40	171
	50–60	6.0	1.03	49
	>60	1.9	0.31	16
Occupational	Farmer	67.4		553
	Migrant workers	16.7		137
	Worker	2.2		18
	Business services	2.2		18
	Student	2.2		18
	Scattered children	1.5		12
	Other	7.9		65

### Space-time clusters

**Fig. 3:**
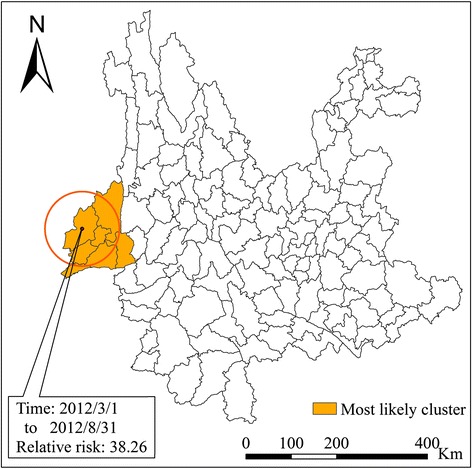
Spatial-temporal clusters of malaria in Yunnan Province, China, setting 50% as the maximum cluster size

In this study, we set different scales of risk population to detect different levels of disease cluster. One significant spatial cluster (O/E = 20.54; *P* < 0.001) was detected between March 1 2012 and August 31 2012 when 50% (which is the default) of the population at risk was set as the size of spatial cluster; this cluster consisted of 390 cases (the rate was 18.52 cases per 10^5^) (Table 2). The cluster, of radius 82.01 km, was detected and centered in the county of Yingjiang in the middle of the western part of Yunnan Province and included the surrounding area; seven counties were involved in total, where the terrain is complex, and the disease crossed the Salween river (Fig. 3).

**Table 2:** Results for the spatial-temporal cluster detection of malaria in Yunnan Province

Category	Cluster	Cluster center	Time: year/month/day	Radius (km)	*P*-value	RR
10%	Most likely	Yingjiang	2012/3/1–2012/8/31	82.01	*P* < 0.001	38.26
	Secondary	Shuangjiang	2012/5/1–2012/7/31	51.99	*P* < 0.001	7.34
		Vesey Lisu	2012/4/1–2012/7/31	67.26	0.0012	7.51
		Yongsheng	2012/5/1–2012/9/30	0.00	0.0440	4.65
30%	Most likely	Yingjiang	2012/3/1–2012/8/31	82.01	*P* < 0.001	38.26
	Secondary	Pu’er	2012/5/1–2012/6/30	143.42	0.0290	2.51
		Yongsheng	2012/5/1–2012/9/30	0.00	0.0700	4.65
50%	Most likely	Yingjiang	2012/3/1–2012/8/31	82.01	*P* < 0.001	38.26

**Fig. 4:**
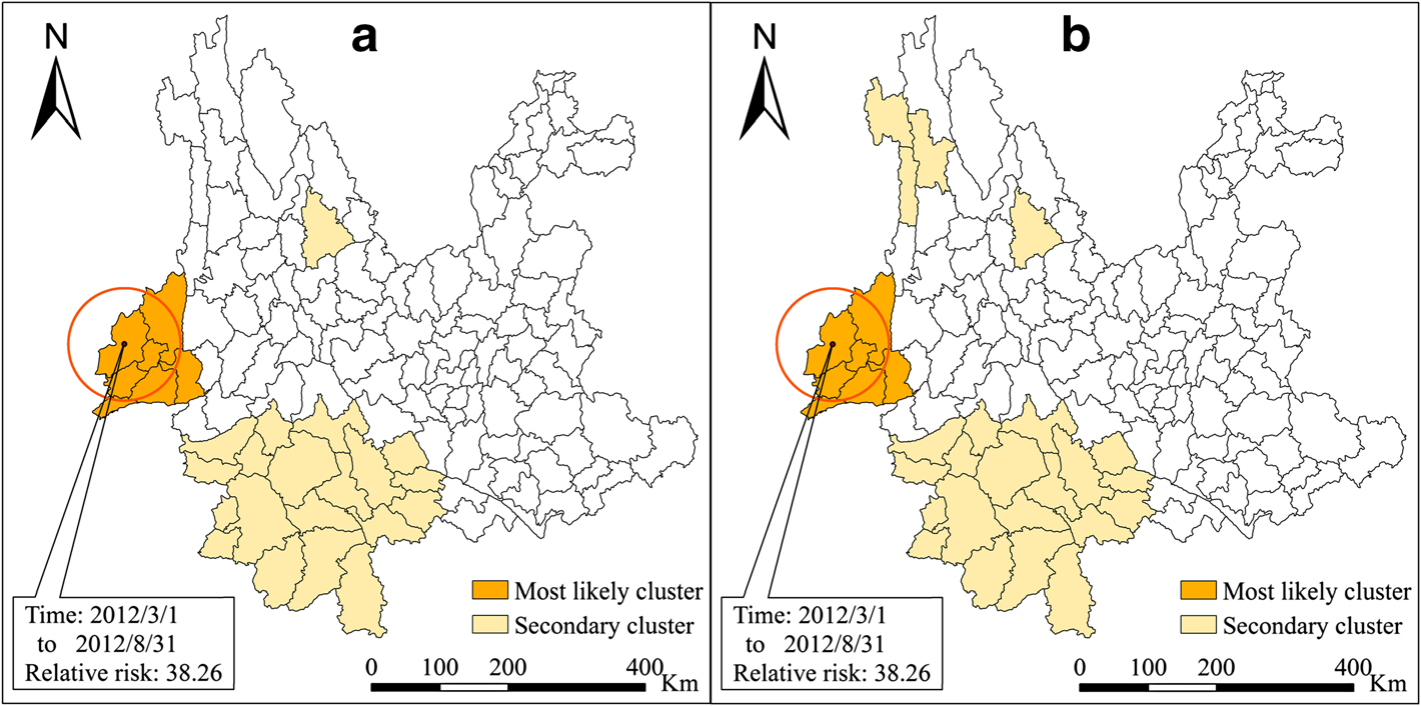
Spatial-temporal clusters of malaria in Yunnan Province, China, setting 30% (**a**) and 10% (**b**) as the maximum cluster size

The maximum size of spatial cluster were modified as 10 and 30% in scan statistic as there might be some small-scale clusters. When the maximum spatial scale was 30% of the population, the cluster which has the highest relative risk (RR) (38.26) was the same as the cluster when the spatial scale was 50%. In addition, two lesser clusters were found: the larger one was centered in Pu’er municipal district, which is located in Southwestern Yunnan, and included 19 geographical districts/counties and a radius of 143.42 km. The time of the cluster was May 1 to June 30 2012, and in this window, the monthly incidence rate was 4.4 per 10^5^ with a RR of 2.51 (*P* = 0.029). And the smaller one centered in the county of Yongsheng, Lijiang city which lay on the upper reach of the Yangtze River and consisted of just one county, Yongsheng. The time of the cluster was May 1 to June 30 in 2012, and the monthly incidence rate in this window was 8.2 per 10^5^ with a RR of 4.65 (*P* = 0.07) (Fig. 4).

When the maximum spatial scale was the lowest, 10% of the total population, several clusters were found. The cluster with the highest RR (38.26) was the same as the cluster when the spatial scale was 50 and 30%. Three secondary clusters were detected, the first centered in the county of Shuangjiang, Lincang city, which is located in Southwestern Yunnan, at the upper reaches of the Mekong river where the Tropic of Cancer crosses; the cluster included four geographic districts/counties with a cluster radius of 51.99 km. The time of the cluster was May 1 to July 31 2012, and in this window, the monthly incidence rate was 12.7 per 10^5^ with a RR of 7.34 (*P* < 0.0001). The second cluster was centered in Vesey Lisu Autonomous County, Diqing Tibetan Autonomous Prefecture, which is located in Northwest Yunnan, where three rivers (Jinsha River, Mekong, Salween) run in parallel. The cluster included three geographical districts/counties, with a cluster radius of 67.26 km. The cluster emerged from April 1 to July 31 2012, and the average monthly incidence rate in this window was 13.3 per 10^5^, with a RR of 7.51 (*P* = 0.0012). The last cluster was the same as the smaller of the secondary clusters when the spatial scale was 30%, and was centered in the county of Yongsheng, Lijiang city (Fig. 4 and Table 2).

### Risk factors

Environmental factors such as meteorological, social and economic conditions, often influence epidemics of disease. Demographic differences, such as the differences in occupational structure may also produce different effects on malaria epidemics in different counties. This article assumes that there are relationships between malaria epidemics and factors such as temperature, precipitation, rice cultivation, and occupational structure (we excluded some factors such as wind speed, humidity, population density, public health conditions, living standards, etc., according to the correlations and multicollinearity found in an exploratory analysis). To examine the roles of environmental factors and occupational structure in malaria epidemics, we selected the annual average temperature (AAT), annual cumulative rainfall (ACR), rice yield per square kilometer (RYPSK) and proportion of rural employees (PRE) as proxy variables for temperature, precipitation, rice cultivation and occupational structure, respectively, and used them as independent variables, with annual malaria incidence as the dependent variable, in constructing a GWR model.

The SAM (Spatial Analysis in Macroecology) software was used to compute the results of parameter estimation and to compare the results of the GWR and OLS regression. The statistical results are presented in Tables 3 and 4. The means and medians were all positive, indicating that AAT, ACR, RYPSK and PRE were positively associated with the malaria incidence rate. The diagnostic statistical results for the GWR and the results of the comparison with OLS regression showed that the GWR was superior than the OLS because it could interpret 52.0% of the total variation with a reduced Akaike Information Criterion (AICc) (F = 13.380, *P* < 0.001).

**Table 3:** Local regression parameter descriptive statistics

Variable	Mean	Minimum	Lwr Quartile	Median	Upr Quartile	Maximum
Constant	−9.630	−24.110	−14.284	−8.057	−4.796	−0.408
AAT (°C)	0.057	−0.234	0.005	0.064	0.087	0.597
ACR (10 mm)	0.090	0.003	0.042	0.075	0.141	0.202
RYPSK (10^3 kg)	0.149	−0.006	0.026	0.100	0.238	0.570
PRE (%)	0.041	−0.090	0.015	0.029	0.063	0.175

**Table 4:** Diagnostic statistics of GWR and OLS

Coefficients	GWR	OLS
AICc	818.469	869.788
Coefficient of Determination (R^2^)	0.557	0.200
Adjusted r-square (R^2^ Adj)	0.520	0.180
F	13.380	7.506
*P*-value	<0.001	<0.001

**Fig. 5:**
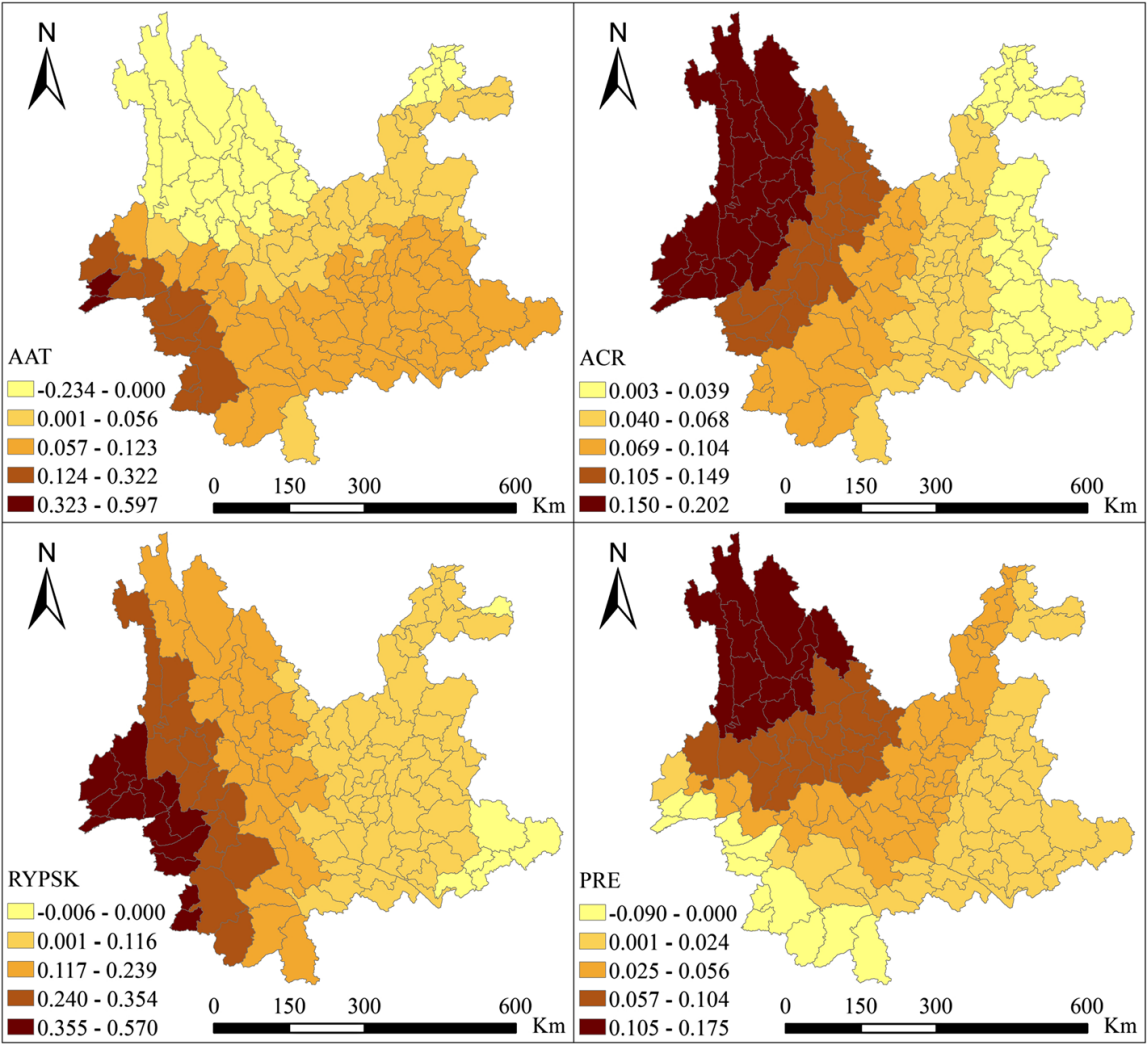
Local regression parameters of AAT, ACR, RYPSK and PRE

**Fig. 6:**
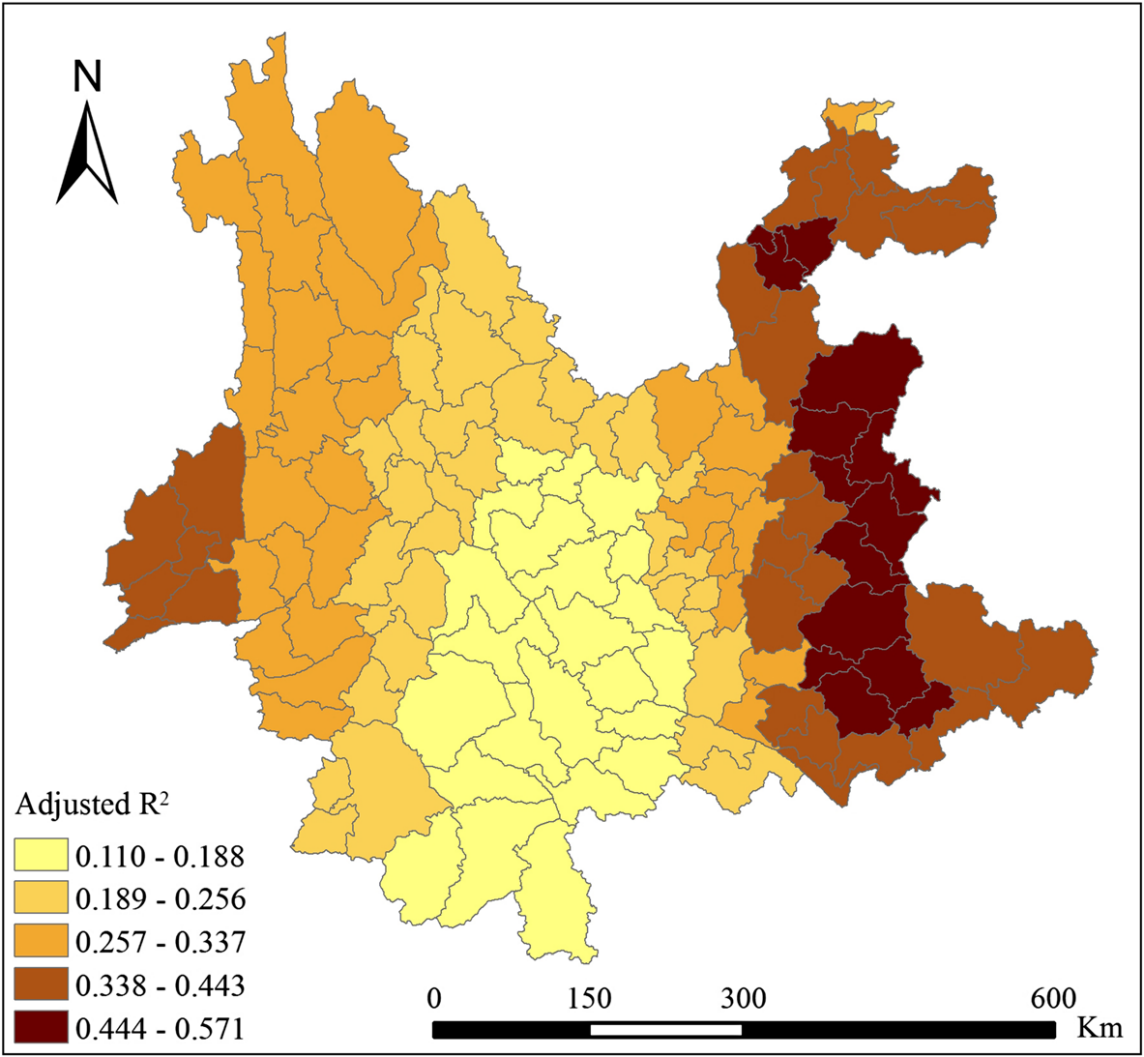
Spatial distribution of Adjusted R^2^

The spatial distributions of the local regression parameters of AAT, ACR, RYPSK and PRE were mapped in order to examine the effects in different regions. The map (Fig. 5) shows that these variables presented different correlations with malaria incidence in different counties. The parameters of AAT presented a high positive correlation with malaria incidence in southwestern counties and a downward trend from south to north. They were reduced to a negative value in northwestern counties with high altitude, indicating that an increase in temperature would decrease the likelihood of malaria epidemics in these counties. The parameters of ACR presented a downward trend from northwest to southeast generally, indicating that its effects on malaria epidemics weakened in this direction, while they showed a positive correlation with malaria incidence throughout the province. The parameters of RYPSK presented a downward trend from west to east and descended into a negative value in several northeastern and southeastern counties, indicating that an increase in rice cultivation was related to malaria epidemics in most counties, but was related to a slight decrease in malaria incidence in several counties in northeastern and southeastern Yunnan province. The parameters of PRE presented a positive correlation with malaria incidence in most counties except for several southwestern counties, indicating that an increase in proportion of rural employees was related to malaria epidemics in most counties, but was related to reduced malaria incidence in several counties in southwestern Yunnan province. The ranges of the parameters of AAT and RYPSK were large (respectively from −0.234 to 0.597 and from −0.006 to 0.570), indicating that the extent of the impacts of temperature and rice cultivation on malaria incidence were large. The spatial distribution of R^2^ (Fig. 6) indicated that the GWR model showed a good explanatory ability in eastern and western counties, but the explanatory ability was poor in counties in south-central Yunnan province.

## Discussion

This study investigated the spatiotemporal epidemic characteristics and risk factors of malaria in Yunnan, an area with a high incidence of malaria in China. The results showed that the group aged 20–30 years had the highest incidence rate, and that men, especially farmers and migrant workers, were the main high-risk groups. Epidemics of malaria were seen in varying degrees of severity during the summer and autumn months, and high-risk regions were mainly distributed in the southwest counties. Temperature, precipitation, rice cultivation and proportion of rural employees were positively associated with the malaria incidence rate generally.

The complex geography is related to the high incidence of malaria in Yunnan. The hot and humid conditions are appropriate for breeding *Anopheles* and other mosquitoes in muggy valleys [23], where the population is concentrated. The epidemiologic characteristics of malaria showed an obvious seasonal pattern and demographic distribution. Summer is the epidemic season of malaria. As shown in Fig. 2, malaria incidence increased rapidly from April and decreased in August, with the peak appearing in June. However, obvious disparities existed in the incidence between males and females, with males contributing to the majority of cases. In these areas, men do more outdoor jobs such as farming and building. They wear less clothing in the hot summer months, and are more likely to be bitten by mosquitoes infected with *Plasmodium* spp. The incidence in the groups aged 20–30, 30–40 and 40–50 years were obviously higher than that in the other age groups. With regard to the occupational distribution, farmers and migrant workers accounted for the majority of cases. To a certain extent, this may be a result of their adverse working conditions.

The results of the SaTScan analysis revealed varying degrees of spatiotemporal clustering in epidemics of malaria in Yunnan Province. The spatiotemporal clusters showed that the high-risk period was concentrated mostly in the summer and autumn months, and the high-risk regions were mainly the southwest counties near the China-Myanmar border, especially in the western counties, where the economic and health conditions are relatively poor and there are more frequent movements of people to Myanmar trading. This results were similar to previous research findings [23–25].

The *Anopheles* mosquito is the natural vector of malaria. Environmental conditions are one of the factors which have significant impacts on the survival rate and the population of *Anopheles* mosquitoes [9, 26]. Thus, there should be an increased risk of infection and higher incidence of malaria in warm and wet conditions. The findings of the GWR model corresponded to the hypotheses on the relationships between environmental conditions and malaria incidence. Generally, annual average temperature, annual cumulative rainfall and rice yield were positively associated with the malaria incidence rate. Previous studies have revealed that a rise in temperature and rainfall is related to increased malaria epidemics [23, 27]. Rice fields near farmhouses provide natural habitats for mosquitoes and increase mosquito densities, and this increases the likelihood of malaria transmission [12, 28–30]. Doing more outdoor jobs, rural employees are prone to be attacked by mosquitoes, increased proportion of rural employees was normally related to malaria epidemics. However, these factors and their combination differed in different regions, which may alter their effects. The GWR results revealed that higher temperatures, precipitation, rice cultivation and proportion of rural employees were all related to increases in the incidence rate of malaria, although raised temperature was generally related to increases in the incidence rate of malaria in most southern counties, raised precipitation and proportion of rural employees were mainly related to increases in the malaria incidence rate in northwestern counties, and raised rice cultivation was mainly related to increases in the malaria incidence rate in western counties. Some researchers have reported similarly that temperature and precipitation have different effects on malaria in different areas and months [9, 31].

These results may be used to inform more targeted control actions. Implementation of malaria interventions during March, before the start of malaria transmission, may improve their efficacy. As identified high risk groups include male farmers and migrant workers, the combined use of control strategies and personal protection against malaria should be implemented during the epidemic season. The results of spatiotemporal cluster detection improve the intervention efficacy from disease prevention department, and the definite risk regions and their space-time informations would certainly benefit disease control strategies. More stringent preventative strategies should be implemented in environments with hot and humid summer and autumn conditions.

There were several limitations of this study that should be noted. First, the research spatial scale was the district/county level in our study, which may have influenced the results of the scanning and factor analysis. The Kulldorff’s SaTScan™ with its scan circle analysis is an effective method in detecting clusters, while the fixed circular window produces defects in examining irregularly polygon and small-scale clusters [32, 33]. Second, the height difference in the terrain is great in this region, which will greatly influence the climatic factors such as temperature and precipitation. However, the differences in meteorological factors within counties were neglected in this study. The research scale which neglected the errors on ecological fallacy may obfuscated some factors [34]. Third, when analyzing the risk factors, we did not consider complex socio-economic factors, which were unavailable for this research, such as living habits (e.g. the use of bed nets) and information on international movement of individuals, which also has an important impact on malaria epidemics [24, 35]. This may be a reason why the GWR could explain only part of the total model (R^2^ = 0.520). Therefore, the relevant socio-economic factors should be investigated and identified in future studies.

## Conclusions

Malaria continues to be one of serious public health issues in Yunnan Province, especially in border counties in southwestern Yunnan. The study involved quantitative analysis of the epidemiological characteristics and space-time clustering of epidemics of malaria in this region. An association between malaria and environmental factors, and occupational structure was revealed; individuals and disease prevention and control departments should implement more stringent preventative strategies in places with hot and humid environmental conditions, to control and eliminate malaria. The quantification and analysis gives a clear sense of the epidemic characteristics of malaria to public health and preventive policy makers, and may contribute to successful national planning of both malaria control and elimination.

## Abbreviations

AAT: Annual average temperature
ACR: Annual cumulative rainfall
AICc: Akaike information criterion
GWR: Geographically weighted regression
LLR: Log likelihood ratio
OLS: Ordinary least squares
PRE: Proportion of rural employees
RR: Relative risk
RYPSK: Rice yield per square kilometer
SAM: Spatial analysis in macroecology
